# WAT1 (WALLS ARE THIN1) defines a novel auxin transporter in plants and integrates auxin signaling in secondary wall formation in Arabidopsis fibers

**DOI:** 10.1186/1753-6561-5-S7-O24

**Published:** 2011-09-13

**Authors:** Philippe Ranocha, Oana Dima, Judith Felten, Amandine Freydier, Laurent Hoffmann, Karin Ljung, Benoit Lacombe, Claire Corratgé, Jean-Baptiste Thibaud, Björn Sundberg, Wout Boerjan, Deborah Goffner

**Affiliations:** 1 Université de Toulouse; UPS; UMR 5546, Surfaces Cellulaires et Signalisation chez les Végétaux; BP 42617, F-31326, Castanet-Tolosan, France; 2 VIB Department of Plant Systems Biology, UGent Department of Plant Biotechnology and Genetics, 9052 Gent, Belgium; 3 Umeå Plant Science Center, Department of Forest Genetics and Plant Physiology, Swedish University of Agricultural Sciences, 90183 Umeå, Sweden; 4 Biochimie et Physiologie Moléculaire des Plantes, CNRS UMR 5004, Institut National de la Recherche Agronomique U386, Montpellier SupAgro, Université Montpellier II, Place Viala, 34060 Montpellier Cedex, France

## Background

Our knowledge of signaling mechanisms involved in secondary cell wall (SCW) formation is quite limited. To discover novel markers of SCW, a genomics approach using *Zinnia elegans* xylogenic cultures was undertaken that identified hundreds of gene candidates expressed at the onset of secondary wall formation [1]. *Arabidopsis* homologs and the corresponding T-DNA mutants for each *Zinnia* gene were identified and the panel of *Arabidopsis* cell wall mutants was subjected to developmental and wall-related phenotyping.

## Results and conclusion

Among the most interesting mutants was *wat1* (*walls are thin1*). The most conspicuous phenotypic feature of *wat1* was the severe reduction (sometimes to the extent of being inexistent) of SCW in xylary and interfascular stem fibers. Interestingly, xylem vessel wall thickness and morphology were not modified by the mutation. In addition to the SCW phenotype, *wat1* was characterized by 5-Me-tryptophan seedling toxicity, severely decreased auxin transport and content in stems, and massive down-regulation of auxin-related genes. These data led us to the conclusion that WAT1 acts as an upstream regulator of SCW deposition in fibers, presumably through an auxin-mediated mechanism [2].

Bioinformatic analysis of WAT1, annotated as ‘homolog to a *Medicago truncatula* nodulin gene, *MtNOD21*, suggested that WAT1 encoded a putative transporter belonging to the Plant Metabolite Exporter family [3]. WAT1:GFP fusion protein experiments localized WAT1 on the tonoplast, confirming the prediction that WAT1 is a membrane protein. Although WAT1 is plant-specific, it shares structural similarities with bacterial amino acid transporters in that it consists of ten transmembrane domains encompassed within a tandem Domain of Unknown Function 6 (DUF6).

To characterize WAT1 function, we recently tested its capacity to transport tryptophan and/or auxin in both yeast and *Xenopus*oocytes. Neither WAT1-expressing yeast cells nor *Xenopus* oocytes were able to facilitate radiolabeled Trp import or export. However, we have been able to demonstrate that WAT1 facilitates auxin import in both expression systems. These results clearly place WAT1 among the ranks, along with PINs, AUX/LAXs andABCB/MDR/PGPs, as a novel, bona fide auxin transporter in plants.

This study constitutes the first functional characterization of any of the 46 members of the WAT1 gene family in *Arabidopsis* and our hope is that this discovery will help pave the way in identifying the functions of other family members. Moreover, the *wat1* mutant will be an ideal tool to address the question as to how auxin subcellular homeostasis plays a role in fiber SCW formation in Arabidopsis. Our current efforts to understand poplar WAT1-mediated auxin signaling in wood formation in trees will also be discussed.
